# Decomposing maternal socioeconomic inequalities in Zimbabwe; leaving no woman behind

**DOI:** 10.1186/s12884-022-04571-9

**Published:** 2022-03-23

**Authors:** Akim Tafadzwa Lukwa, Aggrey Siya, Feyisayo A. Odunitan-Wayas, Olufunke Alaba

**Affiliations:** 1 Health Economics Unit, School of Public Health and Family Medicine, Faculty of Health Sciences, University of Cape Town, Anzio Road, Observatory, Cape Town, 7935 South Africa; 2 DSI-NRF Centre of Excellence in Epidemiological Modelling and Analysis (SACEMA), Stellenbosch University, Private Bag X1, Matieland, Stellenbosch, 7602 South Africa; 3 College of Veterinary Medicine, Animal Resources and Biosecurity, Makerere University, P.O. Box 7062, Kampala, Uganda; 4 Research Centre for Health through Physical Activity, Lifestyle and Sport, Division of Exercise Science and Sports Medicine, Department of Human Biology, Faculty of Health Sciences, University of Cape Town, Cape Town, 7725 South Africa

**Keywords:** Decomposition analysis, Erreygers normalized concentration index, Concentration index, Skilled birth attendance, Antenatal care, Postnatal care, Maternal health in Zimbabwe

## Abstract

**Background:**

Several studies in the literature have shown the existence of large disparities in the use of maternal health services by socioeconomic status (SES) in developing countries. The persistence of the socioeconomic disparities is problematic, as the global community is currently advocating for not leaving anyone behind in attaining Sustainable Development Goals (SDGs). However, health care facilities in developing countries continue to report high maternal deaths. Improved accessibility and strengthening of quality in the uptake of maternal health services (skilled birth attendance, antenatal care, and postnatal care) plays an important role in reducing maternal deaths which eventually leads to the attainment of SDG 3, Good Health, and Well-being.

**Methods:**

This study used the Zimbabwe Demographic Health Survey (ZDHS) of 2015. The ZDHS survey used the principal components analysis in estimating the economic status of households. We computed binary logistic regressions on maternal health services attributes (skilled birth attendance, antenatal care, and postnatal care) against demographic characteristics. Furthermore, concentration indices were then used to measure of socio-economic inequalities in the use of maternal health services, and the Erreygers decomposable concentration index was then used to identify the factors that contributed to the socio-economic inequalities in maternal health utilization in Zimbabwe.

**Results:**

Overall maternal health utilization was skilled birth attendance (SBA), 93.63%; antenatal-care (ANC) 76.33% and postnatal-care (PNC) 84.27%. SBA and PNC utilization rates were significantly higher than the rates reported in the 2015 Zimbabwe Demographic Health Survey. Residence status was a significant determinant for antenatal care with rural women 2.25 times (CI: 1.55–3.27) more likely to utilize ANC. Richer women were less likely to utilize skilled birth attendance services [OR: 0.20 (CI: 0.08–0.50)] compared to women from the poorest households. While women from middle-income households [OR: 1.40 (CI: 1.03–1.90)] and richest households [OR: 2.36 (CI: 1.39–3.99)] were more likely to utilize antenatal care services compared to women from the poorest households. Maternal service utilization among women in Zimbabwe was pro-rich, meaning that maternal health utilization favoured women from wealthy households [SBA (0.05), ANC (0.09), PNC (0.08)]. Wealthy women were more likely to be assisted by a doctor, while midwives were more likely to assist women from poor households [Doctor (0.22), Midwife (− 0.10)].

**Conclusion:**

Decomposition analysis showed household wealth, husband’s education, women’s education, and residence status as important positive contributors of the three maternal health service (skilled birth attendance, antenatal care, and postnatal care) utilization outcomes. Educating women and their spouses on the importance of maternal health services usage is significant to increase maternal health service utilization and consequently reduce maternal mortality.

## Background

Maternal health undoubtedly remains an important global health priority [1]. The Sustainable Development Goals (SDGs) argue for not leaving anyone behind in attaining these goals [2]. Specifically, SDG 3.1 aims to “reduce the global maternal mortality ratio to less than 70 per 100 000 live births by 2030” [2]. Recently, maternal health estimates reflect that about 830 women die from pregnancy or childbirth-related complications around the world daily [3]. The global maternal mortality ratio was 152 deaths per 100,000 live births in 2020, reflecting an increase from 151 deaths per 100,000 live births reported in 2019 [4]. This trajectory projects 133 deaths per 100,000 live births in 2030, nearly double the SDG target. Most of these maternal deaths occur in low-resource settings and could be avoided [5], with timely and proper use of; antenatal care (ANC), skilled birth attendance (SBA), and postnatal care (PNC) [1, 6–20].

Globally, the attainment of low maternal mortality rates is under great threat from; growing socio-economic inequalities, poor health services, political unwillingness (minimal government effort), and cultural constraints [6]. Reasonable evidence from literature reported socioeconomic inequalities to be high in developing countries, whose health systems are under-developed [1, 10–12, 21]. In most cases, health inequalities are argued to be affecting people of low socioeconomic status disproportionately. The healthy and wealthy are more likely to obtain health care when compared to the sick and the poor [22]. Sub-Saharan African (SSA) health systems are reported to have socioeconomically unevenly distributed health outcomes and access to key health services. For instance, it has been documented in the literature that women from socioeconomically disadvantaged households experience higher morbidity, mortality rates, and lower coverage of maternal health services than those from wealthier households [23–26].

Despite the growing literature on maternal health across Sub-Saharan African countries, it is unfortunate that relatively little is known on the evolution of socioeconomic inequalities over time. Even though it is imperative to note that several studies have been done on maternal health inequalities [26–28], to our knowledge no study has yet decomposed the socioeconomic inequalities to assess what is driving the maternal health inequalities in Zimbabwe. Given that key health care interventions are essential in reducing and preventing deaths due to pregnancy-related causes [29], adherence to prenatal care, delivering in health facilities, and having a skilled health worker at delivery could improve maternal health. This study assessed socioeconomic inequalities in the uptake of maternal health services in Zimbabwe, by assessing socioeconomic indicators across three maternal health indicators (skilled birth attendance, antenatal care, and postnatal care) using the latest available 2015 Zimbabwe Demographic Health Survey.

## Methods

### Data sources & study population

The study used secondary data from Zimbabwe’s Demographic Health Surveys (ZDHS) of 2015. The 2015 ZDHS population sample was nationally representative, comprising of more than 11,000 households [30, 31]. The 2015 ZDHS was representative of each of Zimbabwe’s ten provinces: Manicaland, Mashonaland Central, Mashonaland East, Mashonaland West, Matabeleland North, Matabeleland South, Midlands, Masvingo, Harare, and Bulawayo. The 2015 ZDHS used the 2012 sampling frames [30, 31]. The 2015 Demographic Health Survey used a two-stage cluster sampling approach; in the first stage, the samples included 2015 400 Enumeration Areas (EAs), that is, 166 in urban areas and 234 in rural areas. The second stage of sampling included a complete listing of households conducted for each of the selected 400 Enumeration Areas (EAs) in March 2015, respectively [30, 31]. The study population was composed of women of child-bearing age (15–49 years) interviewed in 2015. The sample retained from the 2015 ZDHS before taking into account some observations with missing data on variables of interest was 9955 women. However, after including only observations that had full records on variables of interest the study sample reduced to 4595 women.

### Statistical analysis

This study employed 3 statistical analyses namely; logistic regression, Erreygers Normalised concentration indices, and decomposition of the Erreygers Normalised concentration indices. The logistic regression models were used to estimate the likelihood of uptake of maternal health services (SBA,ANC & PNC) among women aged 15 to 49. When using logistic regression, the odds ratios were determined for all independent variables for each category of the independent variable with the exception of the reference category, which was used as a reference category in the analysis. After assessing the association of maternal health services uptake with the demographics variables, we estimated health inequalities in maternal health uptake as well as what was driving the health inequalities in Zimbabwe using the Erreygers Normalised concentration indices. We used the output of the logistic regression in developing and decomposing the Erreygers Normalised concentration indices. The Erreygers Normalised concentration indices are explained in detail under the concentration curves sub-heading.

### Outcome variables

Maternal health in this study was measured using 3 outcome variables thus; skilled birth attendance, antenatal care, and postnatal care. Outcome variables were categorized into binary variables: Skilled birth attendance was assigned a value of 1 if a woman reported being attended by a doctor, nurse, or midwife during delivery, and 0 if otherwise. Antenatal care in this study was, defined as mothers who received pregnancy care from skilled health providers (doctors, nurses, and nurse midwives) [31], and represented by 1 if a woman had received at least four ANC visits and 0 for less than four ANC visits. Lastly, as safe motherhood programs recommend that women receive a postnatal health check within 2 days after delivery [31], for this study postnatal care was reported on mothers who had received a postnatal check in the first 2 days after delivery and coded as 1 for mothers who has received postnatal care, and 0 otherwise.

### Selection of regressor variables

Socioeconomic factors such as women’s age, women’s education, partner’s education, residence status, household wealth, household head sex, employment status, place of delivery, antenatal care, postnatal care, birth order, distance to the health facility, and media access (radio/television) have been widely reported as key determinants of inequalities in maternal health care uptake [1, 7, 10–20, 23–26, 32, 33]. This study then used the aforementioned determinants as predictors in the regression models.

### Analysis of the association of the predictors with the outcome variables

The study computed binary logistic regressions to predict the dependant variables: skilled birth attendance, antenatal care, and postnatal care. Binary logistic regression is known to be most useful when the dependent variable is a dichotomous [34]. Women’s and partners’ education were both categorized into four groups;0 no education, 1 primary, 2 secondary, and 3 tertiary education. Residence status was categorized into 2 groups and coded as; 0 urban and 1 rural. Birth order was grouped into 4 groups;1st,2nd, 3rd and 4^+^. Women’s age was grouped into 4 categories namely 15–24, 25–34, 35–44, and 45–49 years.

### Socioeconomic status

The wealth index was retained as it was in the Demographic Health Survey [30, 31]. In the ZDHS survey, the household wealth index was calculated by constructing a linear index from asset ownership indicators using principal components analysis to derive weights [30, 31]. In the original survey, the wealth index was constructed by assigning household scores, then ranking each person in the household population by their score. Thereafter, the distribution was divided into five equal categories and each had 20% of the population with economic proxies, such as housing quality, household amenities, consumer durables, and size of landholding [30, 31]. This study then retained the wealth index as recorded in the original survey 5 groups (poorer, poor, middle, richer, richest). This study adopted the household wealth index as a proxy for a household’s economic status.

### Concentration curves and indices

The concentration index approach is a standard measure of assessing health inequalities. The indices and curves investigate whether the health inequalities exist in one group or not. However, they do not estimate the magnitude of the health inequalities [35]. This paper used the Erreygers normalized concentration indices [36], to measure the degree of socioeconomic inequalities in utilization of antenatal care, postnatal care, and skilled birth attendance services in Zimbabwe. Among many of the indices that could have been used, we opted to adopt the Erreygers due to its ability to be decomposable.

The concentration index can be computed making use of the ‘covariance’ as shown below:

1 $$CI=\frac{2}{\hat{y}} COV\ \left({y}_i,{R}_i\right)$$

Where: y_i_ is the health variable.

ŷ is the mean of y_i_.

R_i_ is the fractional rank of the ith individual.

COV denotes the covariance.

Concentration indices can be computed as twice the area between the concentration curve and the line of equality (the 45-degree line) [37]. No existence of health inequality is reflected by a concentration curve lying on the 45° line. The extent of the health inequality is shown by how far the concentration curve lies away from the line of equality (45° line). The further the concentration curve is from the line of equality, the greater the extent of health inequality [35]. Therefore, a true zero value of the Erreygers normalized concentration index indicates no existence of socioeconomic inequalities, while a negative value translates to the disproportionate concentration of socioeconomic inequalities among the poor and a positive value reflects the concentration of socioeconomic inequalities among the rich [9, 38].

Since skilled birth attendance, antenatal care, and postnatal care were cardinal variables, as the differences between health states were comparable, the study adopted the Erreygers normalized index (E(c)). The study opted to use the normalized formulae as, [36, 39] argued that normalization of the health concentration index formula ensured remedying the bounds issue for binary cardinal health variables. The Erreygers normalized index (E(c)) can be expressed as:

2 $${E}_c=\frac{4\hat{y}}{y^{max}-{y}^{min}} CI$$

Where y^max^ - y^min^ is the range of the health variable, which i**s** ‘one’ in the case of binary variables. Given that both corrected CIs are commonly used in the health literature, the present study focused on the Erreygers normalised index.

### Decomposing the Erreygers normalised concentration index

The Erreygers Normalised concentration index is decomposable, so as to compute the contributions of determinants of maternal health indicators [40, 41]. Health inequalities were decomposed into the contributions of various explanatory factors, with each contribution as the product of the elasticity of health. Assuming a linear relationship between individual health (y_i_) and a set of k explanatory variables y_i_ will be:

3 $${y}_i=a+\sum_k{\beta}_k{X}_{ki}+{\varepsilon}_i$$

Wagstaff et al. showed that for any health variables exhibiting a linear relationship with a set of k exploratory variables, the concentration index for the health variable can be decomposed as follows:

4 $$CI=\sum_k\left(\frac{\beta_k{\dot{x}}_k}{\hat{y}}\right){CI}_k+\frac{GCI_{\varepsilon }}{\hat{y}}$$

Where: β_k is_ the partial.

ŷ is the mean of the health variable (SBA or ANC or PNC).

ẋ_k_ is the mean of ẋ_k_.

CI_k_ denotes the concentration index of x_k_ against Wealth index/Socioeconomic Status.

GC_ɛ_ is the generalized concentration for the error term.

Equation (4) can be modified as shown below to decompose the Erreygers concentration index [42]

5 $${E}_c=4\left[\sum_k\left({\beta}_k{\dot{x}}_k\right){CI}_k+{GCI}_{\varepsilon}\right]$$

## Results

### Descriptive statistics

Overall affirmative response for maternal health utilization was skilled birth attendance (SBA), 93.63%; antenatal care (ANC) 76.33% and postnatal care (PNC) 84.27%. Maternal health utilization was highest among 24–34-year old’s [skilled birth attendance SBA (48.89%), antenatal care ANC (49.04%), postnatal care PNC (48.82%)] (Table 1). As expected, older women (less than 1.5%) used maternal health services the least compared to all other age groups (45–49-year old’s; SBA (1.04%), ANC (1.09%), PNC (0.98%) [Table 1]. Rural women utilized maternal health services more than urban women [SBA (65.76%) vs (34.24%), ANC (65.99%) vs (34.01%), PNC (65.28%) vs (34.72%)] (Table 1).

**Table 1:** Maternal health utilisation by socio-demographic characteristics

Socio-demographic	SBA^a^ utilisation N (%)	Chi-square	ANC^b^ utilisation N (%)	Chi-square	PNC^c^ utilisation N (%)	Chi-square
***Women age groups***						
15-24 years	1348 (30.46)	0.91	1070 (29.67)	0.03	1208 (30.33)	0.41
25-34 years	2163 (48.89)		1769 (49.04)		1944 (48.82)	
35-44 years	868 (19.61)		728 (20.20)		791 (19.87)	
45-49 years	45 (1.04)		39 (1.09)		38 (0.98)	
***Residence status***						
Urban	1515 (34.24)	0.00	1226 (34.01)	0.00	1382 (34.72)	0.00
Rural	2909 (65.76)		2380 (65.99)		2600 (65.28)	
***Women’s education***						
No education	48 (1.11)	0.00	41 (1.14)	0.00	40 (1.02)	0.00
Primary	1278 (28.88)		1050 (29.12)		1109 (27.85)	
Secondary	2845 (64.30)		2287 (63.41)		2595 (65.16)	
Tertiary	252 (5.72)		228 (6.33)		237 (5.97)	
***Partner’s education***						
No education	40 (1.09)	0.00	35 (1.15)	0.00	38 (1.15)	0.00
Primary	776 (20.80)		641 (20.93)		670 (19.96)	
Secondary	2561 (68.64)		2074 (67.63)		2329 (69.31)	
Tertiary	353 (9.47)		315 (10.29)		322 (9.58)	
***Socioeconomic status***						
Poorest	909 (20.55)	0.00	723 (20.07)	0.00	794 (19.95)	0.00
Poorer	818 (18.50)		648 (17.98)		719 (18.07)	
Middle	764 (17.28)		652 (18.08)		703 (17.67)	
Richer	1068 (24.15)		828 (22.98)		988 (24.82)	
Richest	863 (19.52)		753 (20.89)		776 (19.50)	
***Household head sex***						
Male	2800 (63.28)	0.06	2286 (63.38)	0.67	2497 (62.71)	0.63
Female	1624 (36.72)		1321 (36.62)		1485 (37.29)	
***Employment status***						
Unemployed	2101 (47.49)	0.66	1685 (46.72)	0.02	1843 (46.29)	0.00
Employed	2323 (52.51)		1922 (53.28)		2139 (53.71)	
***Delivery place***						
Home	615 (13.92)	0.00	440 (12.21)	0.00	539 (13.54)	0.00
Public hospitals	1681 (38.00)		1393 (38.62)		1515 (38.05)	
Public clinics	1546 (34.95)		1244 (34.50)		1417 (35.58)	
Private hospitals/clinics	249 (5.64)		238 (6.61)		222 (5.58)	
Mission hospitals/clinics	331 (7.50)		290 (8.06)		288 (7.25)	
***Antenatal care***						
Less than 4 ANC visits	825 (18.66)	0.00			800 (20.11)	0.00
At least 4 ANC visits	3599 (81.34)				3181 (79.89)	
***Postnatal care***						
Not receive PNC	558 (12.61)	0.00	425 (11.80)			
Received PNC	3867 (87.39)		3181 (88.20)			
***Birth order***						
1st	1111 (25.12)	0.00	899 (24.94)	0.00	1015 (25.49)	0.01
2nd	1122 (25.36)		924 (25.63)		988 (24.82)	
3rd	946 (21.40)		772 (21.40)		846 (21.26)	
4^+^	1244 (28.12)		1010 (28.02)		1132 (28.43)	
***Distance to health facility***						
Not a big problem	2820 (63.73)	0.00	2315 (64.18)	0.00	2558 (64.23)	0.00
A big problem	1605 (36.27)		1292 (35.82)		1424 (35.77)	
***Radio access***						
Not at all	1840 (41.59)	0.00	1490 (41.31)	0.01	1620 (40.68)	0.00
Less than once a week	959 (21.69)		765 (21.22)		887 (22.29)	
At least once a week	1625 (36.73)		1351 (37.47)		1474 (37.03)	
***Television access***						
Not at all	2573 (58.14)	0.00	2055 (56.97)	0.00	2291 (57.53)	0.00
Less than once a week	597 (13.51)		501 (13.91)		549 (13.80)	
At least once a week	1254 (28.35)		1050 (29.12)		1141 (28.67)	

^a^Skilled Birth Attendance

^b^Antenatal Care

^c^Postnatal Care

Maternal services utilization was highest among secondary educated women [SBA (64.30%), ANC (63.41%), PNC (65.16%)], with secondary educated partners [SBA (68.64%), ANC (67.63%), PNC (69.31%)] (Table 1). Maternal service utilization was least in households with uneducated women [SBA (1.11%), ANC (1.14%), PNC (1.02%)], with uneducated partners [SBA (1.09%), ANC (1.15%), PNC (1.15%)] (Table 1). There was no significant difference in maternal services utilization across socioeconomic groups. Skilled birth attendance (SBA) and postnatal care (PNC) utilization were highest among richer women [SBA (24.15%), PNC (24.50%)] and lowest among women from middle-income households [SBA (17.28%), PNC (17.67%)] (Table 1). However, antenatal care (ANC) utilization was highest among richer women (22.98%) and lowest among poorer women (17.98%) [Table 1].

Maternal services utilization was highest among male-headed households [SBA (63.28%), ANC (63.38%), PNC (62.71%)] (Table 1). Surprisingly, maternal services utilization was highest in public hospital [SBA (38.00%), ANC (38.62%), PNC (38.05%)] and least in private hospitals/clinics [SBA (5.64%), ANC (6.61%), PNC (5.58%)] (Table 1). Skilled birth attendance (81.34%) and postnatal care (79.89%) utilization were highest among women who had attained at least four antenatal care visits, while postnatal care utilization was highest among women who had received skilled birth attendance (87.39%) and attained at least four antenatal care visits (88.20%) [Table 1]. There were no significant differences in maternal services utilization by birth order, however, distance to health facility showed significant variations in maternal services utilization. As expected, utilization was high among those who viewed distance to a health facility as not a big problem [SBA (63.73%), ANC (64.18%), PNC (64.23%)] (Table 1). However, media access presented astonishing maternal services utilization rates in Zimbabwe. Maternal services utilization was highest among women who had no radio [SBA (41.59%), ANC (41.31%), PNC (40.68%)] and television [SBA (58.14%), ANC (56.97%), PNC (57.53%)] access compared to those who accessed both less than once a week and at least once a week.

### Socioeconomic determinants of maternal healthcare utilization

All models were statistically significant, [SBA; LR chi2 (**30**) = 1209.36, *p* < 0.00), ANC; LR chi2 (**30**) = 916.54, *p <* 0.00, PNC; LR chi2 (**30**) = 530.92, *p <* 0.00] reflecting that the models were able to distinguish between those who reported maternal service utilization as good and vice versa. Women’s age was only a significant predictor for skilled birth attendance utilisation and postnatal care utilisation in Zimbabwe at 95% confidence interval (CI). With 24–34 [OR: 2.32 (CI: 1.28–4.20)] and 35–44 [OR: 4.65 (CI: 2.10–10.29)] year olds more likely to utilize skilled birth attendance services compared to 15-24 year olds (Table 2). While, 35–44 year olds were more likely to utilize postnatal care services compared to 15–24 year olds (Table 2). Residence status was only a significant determinant for antenatal care and rural women were 2.25 (CI: 1.55–3.27) times more likely to utilize antenatal care services compared to urban women (Table 2).

**Table 2:** Logistic regression results for maternal health services^1^

	Skilled Birth Attendance		Antenatal Care		Postnatal Care	
Socio-demographic Characteristics	*Odds Ratio [Conf. Interval*^2^*]*	*Standard Error*	*Odds Ratio [Conf. Interval]*	*Standard Error*	*Odds Ratio [Conf. Interval]*	*Standard Error*
***Women age groups***						
15-24 years	ref	ref	ref	ref	ref	ref
25-34 years	2.32^a^ [1.28 4.20]	0.70	1.06 [0.82 1.38]	0.14	1.17 [0.88 1.55]	0.17
35-44 years	4.65^a^ [2.10 10.29]	1.89	1.26 [0.89 1.80]	0.23	1.51^b^ [1.03 2.21]	0.30
45-49 years	2.57 [0.33 20.01]	2.69	1.56 [0.64 3.82]	0.71	1.21 [0.52 2.81]	0.52
***Residence status***						
Urban	ref	ref	ref	ref	ref	ref
Rural	0.64 [0.27 1.49]	0.28	2.25^a^ [1.55 3.27]	0.43	1.06 [0.72 1.57]	0.21
***Women’s education***						
No education	ref	ref	ref	ref	ref	ref
Primary	0.08^b^ [0.01 0.82]	0.10	0.78 [0.35 1.76]	0.32	0.92 [0.42 2.00]	0.36
Secondary	0.12 [0.01 1.19]	0.14	0.60 [0.27 1.37]	0.25	1.28 [0.58 2.82]	0.52
Tertiary	0.32 [0.00 33.69]	0.76	0.80 [0.29 2.18]	0.41	2.06 [0.74 5.70]	1.07
***Partner’s education***						
No education	ref	ref	ref	ref	ref	ref
Primary	5.17^b^ [1.17 22.77]	3.91	0.77 [0.31 1.91]	0.36	0.55 [0.23 1.34]	0.25
Secondary	4.30^b^ [1.00 18.73]	3.23	0.68 [0.27 1.68]	0.31	0.59 [0.24 1.43]	0.27
Tertiary	8.01^b^ [1.02 62.81]	8.42	0.75 [0.28 2.00]	0.38	0.55 [0.21 1.48]	0.28
***Socioeconomic status***						
Poorest	Ref	ref	ref	ref	ref	ref
Poorer	1.07 [0.61 1.86]	0.30	0.97 [0.74 1.26]	0.13	0.88 [0.67 1.16]	0.12
Middle	0.56 [0.30 1.08]	0.19	1.40^b^ [1.03 1.90]	0.22	1.21 [0.87 1.67]	0.20
Richer	0.20^a^ [0.08 0.50]	0.09	1.17 [0.78 1.76]	0.24	0.90 [0.59 1.37]	0.20
Richest	0.64 [0.16 2.66]	0.47	2.36^a^ [1.39 3.99]	0.63	0.97 [0.55 1.73]	0.29
***Household head sex***						
Male	ref	ref	ref	ref	ref	ref
Female	0.49^a^ [0.32 0.74]	0.10	0.89 [0.74 1.07]	0.08	0.97 [0.79 1.20]	0.10
***Employment status***						
Unemployed	ref	ref	ref	ref	ref	ref
Employed	0.67^a^ [0.45 1.00]	0.14	1.10 [0.92 1.31]	0.10	1.26^b^ [1.03 1.53]	0.13
***Delivery place***						
Home	ref	ref	ref	ref	ref	ref
Public hospitals	13.73^a^ [7.67 24.58]	4.08	2.27^a^ [1.74 2.95]	0.31	2.89^a^ [2.19 3.80]	0.41
Public clinics	17.44^a^ [9.47 32.12]	5.43	1.90^a^ [1.47 2.45]	0.25	3.76^a^ [2.86 4.95]	0.53
Private hospitals/clinics	17.03^b^ [1.08269.45]	23.99	7.64^a^ [3.64 16.05]	2.89	1.87^b^ [1.08 3.21]	0.52
Mission hospitals/clinics	45.71^a^ [5.64370.37]	48.79	2.88^a^ [1.89 4.37]	0.62	2.17^a^ [1.46 3.24]	0.44
***Antenatal care***						
Less than 4 ANC visits	ref	ref			ref	ref
At least 4 ANC visits	100.06^a^ [45.84218.41]	39.85			1.30^b^ [1.02 1.66]	0.16
***Postnatal care***						
Not receive PNC	Ref	ref	ref	ref		
Received PNC	4.65^a^ [3.10 6.96]	0.96	1.30^b^ [1.02 1.65]	0.16		
***Birth order***						
1st	ref	ref	ref	ref	ref	ref
2nd	0.95 [0.48 1.87]	.3276241	0.89 [0.67 1.18]	0.13	0.62^a^ [0.45 0.85]	0.10
3rd	0.55 [0.25 1.19]	0.22	0.82 [0.60 1.13]	0.14	0.70^b^ [0.48 1.01]	0.13
4^+^	0.20^a^ [0.09 0.45]	0.08	0.73 [0.51 1.03]	0.13	0.59^a^ [0.40 0.87]	0.12
***Distance to health facility***						
Not a big problem	ref	ref	ref	ref	ref	ref
A big problem	1.27 [0.81 1.98]	0.29	1.03 [0.84 1.25]	0.10	1.02 [0.82 1.27]	0.11
***Radio access***						
Not at all	ref	ref	ref	ref	ref	ref
Less than once a week	1.07 [0.64 1.80]	0.28	0.79^b^ [0.63 1.00]	0.09	1.42^a^ [1.08 1.85]	0.19
At least once a week	2.00^a^ [1.25 3.21]	0.48	1.00 [0.82 1.23]	0.11	1.21 [0.97 1.50]	0.14
***Television access***						
Not at all	ref	ref	ref	ref	ref	ref
Less than once a week	1.68 [0.82 3.46]	0.62	1.48^a^ [1.11 1.98]	0.22	1.20 [0.86 1.67]	0.20
At least once a week	1.21 [0.65 2.27]	0.3	1.28^b^ [0.99 1.66]	0.17	1.04 [0.77 1.41]	0.16
***Skilled birth attendance***						
Did not receive SBA			ref	ref	ref	ref
Received SBA			80.68^a^ [38.47169.18]	30.48	4.82^a^ [3.38 6.88]	0.87

^1^a and b indicate statistical significance at the 1 and 5%, respectively

^2^Confidence Interval

Women’s and partner’s education were only significant determinants for skilled birth attendance at 95% confidence interval (CI). With primary educated women less likely to use skilled birth attendance services [OR: 0.08 (CI: 0.01–0.82)] compared to uneducated women (Table 2). Women who had educated partners {primary [OR: 5.17 (CI: 1.17–22.77)], secondary [OR: 4.30 (CI: 1.00–18.73)] and tertiary [OR: 8.01 (CI: 1.02–62.81)]} were more likely to utilize skilled birth attendance services compared to women with uneducated partners (Table 2).

Household wealth was a significant predictor for skilled birth attendance and antenatal care at 95% confidence interval. Richer women were less likely to utilize skilled birth attendance service [OR: 0.20 (CI: 0.08–0.50)] compared to women from the poorest households (Table 2). While, women from middle income households [OR: 1.40 (CI: 1.03–1.90)] and richest households [OR: 2.36 (CI: 1.39–3.99)] were more likely to utilize antenatal care services compared to women from the poorest households (Table 2). Household head sex was a significant predictor of skilled birth attendance only, with female headed households less likely to utilize skilled birth attendance services [OR: 0.49 (CI: 0.32–0.74)] (Table 2). While, employment status was only a significant predictor in the utilization of skilled birth attendance and postnatal care, with employed women less likely to utilize skilled birth attendance [OR: 0.67 (CI: 0.45–1.00)], however, employed women were more likely to utilize postnatal care services [OR: 1.26 (CI: 1.03–1.53)] (Table 2).

Place of delivery was a significant determinant of maternal services utilization in Zimbabwe. Women delivering at; public clinic and hospitals, private hospitals/clinics and mission hospitals/clinics were more likely to utilize SBA, ANC and PNC services than those delivering at home (Table 2). Antenatal care was a significant determinant for skilled birth attendance (SBA) and postnatal care (PNC) utilization. Women who had attained at least four antenatal care visits were more likely to utilize SBA [OR: 100.06 (CI: 45.84–218.41)] and PNC [OR: 1.30 (CI: 1.02–1.66)] (Table 2). Receiving PNC was a significant determinant for skilled birth attendance [OR: 4.65 (CI: 3.10–6.96)] and antenatal care [OR: 1.30 (CI: 1.02–1.65] (Table 2). Women who had radio access at least once week [OR: 2.00 (CI: 1.25–3.21)] were more likely to utilize skilled birth attendance services compared with women with no radio access at all. Women who had radio access for less than once a week were less likely to utilize antenatal care services [OR: 0.79 (CI: 0.63–1.00)] and also women who had radio access less than once week [OR: 1.42 (CI: 1.08–1.85)] were more likely to utilize postnatal care services than those with no radio access (Table 2). Women who received skilled birth attendance were more likely to utilize antenatal care [OR: 80.68 (CI: 38.47–169.18)] and postnatal care [OR: 4.82 (CI: 3.38–6.88)] services (Table 2).

### Concentration indices

**Fig. 1:**
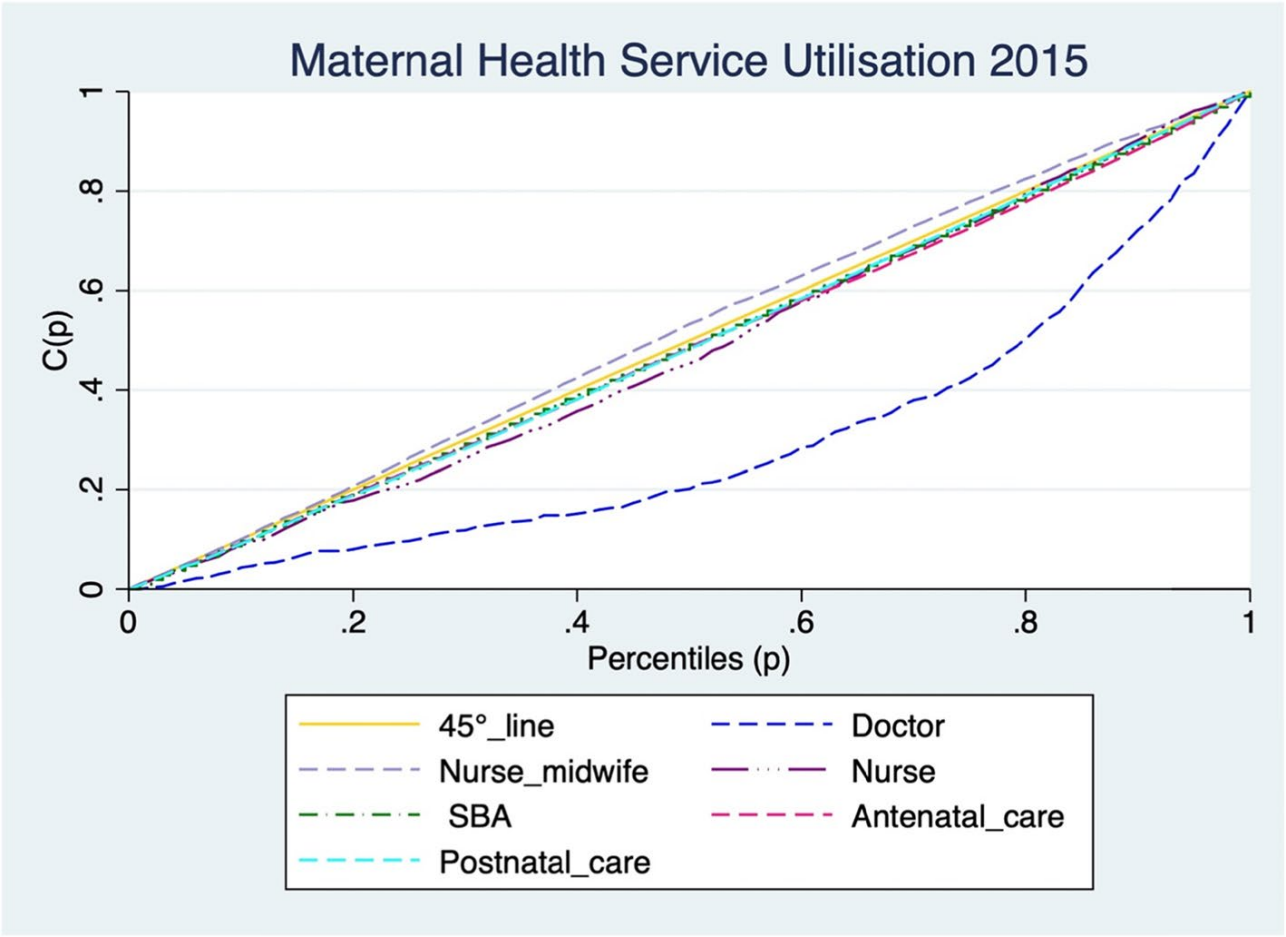
Maternal health utilisation concentration curves for 2015

Maternal service utilization among women in Zimbabwe was pro-rich, meaning that maternal health utilization favoured women from wealthy households [SBA (0.05), ANC (0.09), PNC (0.08)]. The study further, assessed concentration indices of skilled birth attendance by health personnel, however, only those assisted by the doctor and midwife had concentration indices that were significant (*p <* 0.05).^1^ Wealthy women were more likely to be assisted by a doctor, while midwives were more likely to assist women from poor households [Doctor (0.22), Midwife (− 0.10)] (Table 3). The concentration curves drawn concurred with the concentration indices (Fig. 1). As the concentration curves of SBA,ANC,PNC and doctor assisted crossed or were tangent to the 45° line, dominance tests were computed against the line of equality and all tests showed non-dominance.

**Table 3:** Erreygers normalised indices of maternal health services in Zimbabwe^1^

***Characteristic***	***Erreygers normalized index***	***Robust Standard errors***
Skilled birth attendance	0.05^a^	0.01
Doctor assisted	0.22^a^	0.02
Midwife assisted	-0.10^a^	0.02
Nurse assisted	0.04	0.02
Antenatal care	0.09^a^	0.02
Postnatal care	0.08^a^	0.02

^1^a and b indicate statistical significance at the 1 and 5%, respectively; Standard. Errors were adjusted for 399 clusters in the primary sampling unit

### Erreygers decomposition

The study decomposed the Erreygers concentration (E_c_) indices to understand the contribution (Contri) of socio-demographic factors to health inequalities of maternal service utilisation in Zimbabwe. Residence status (Contri: 13.69%, E_c_: − 0.32), women’s education (Contri: 12.79%, E_c_: − 0.09), household wealth (Contri: 38.76%, E_c_: 0.27), antenatal care (Contri: 13.69%, E_c_: − 0.32) and television access (Contri: 13.69%, E_c_: − 0.32) were positive significant contributors, while, place of delivery (Contri: − 27.28%, E_c_: 0.07) was a negative significant contributor to maternal health inequalities in skilled birth attendance in Zimbabwe (Table 4).

**Table 4:** Decomposition of maternal health services by socio-demographic characteristics

	***Skilled birth attendance***				***Antenatal Care***				***Postnatal Care***			
	Elasticity	Concentration Index	Absolute Contri^a^.	% Contri.	Elasticity	Concentration Index	Absolute Contri.	% Contri.	Elasticity	Concentration Index	Absolute Contri.	% Contri.
Women’s age	0.00	0.01	0.00	0.32	0.01	0.01	0.00	0.32	0.00	0.01	0.00	0.05
Residence status	−0.01	−0.32	0.01	13.69	0.08	−0.32	−0.10	−110.00	0.00	−0.32	0.00	−3.38
Women’s Education	0.02	0.09	0.01	12.79	− 0.02	0.09	−0.01	7.93	0.07	0.09	0.03	32.53
Partner’s Education	0.01	0.09	0.00	−3.46	0.00	0.09	0.00	−1.35	− 0.01	0.09	0.00	−3.73
Household Wealth	−0.02	0.27	− 0.02	38.76	0.09	0.27	0.10	106.53	0.02	0.27	0.03	32.49
Household head sex	−0.01	− 0.02	0.00	0.71	0.00	−0.02	0.00	0.32	0.00	− 0.02	0.00	−0.24
Employment status	−0.01	0.12	0.00	6.47	0.01	0.12	0.00	3.57	0.01	0.12	0.01	6.93
Delivery place	0.06	0.06	0.01	−27.28	0.05	0.06	0.01	12.37	0.04	0.06	0.01	12.05
Antenatal care	0.10	0.03	0.01	23.75					0.03	0.03	0.00	4.29
Postnatal care	0.04	0.03	0.00	8.54	0.03	0.03	0.00	4.00				
Distance to facility	0.00	−0.32	0.00	7.30	0.00	−0.32	0.00	−1.92	0.00	−0.32	0.00	2.01
Radio access	0.01	0.08	0.00	−5.55	0.00	0.08	0.00	−1.65	0.01	0.08	0.00	4.75
Television access	0.00	0.47	0.01	17.00	0.02	0.47	0.03	35.93	0.00	0.47	0.00	0.17
Skilled birth attendance					0.59	0.02	0.04	43.87	0.20	0.02	0.01	17.98
***Residuals***	***6.94%***				***0.07%***				***−5.90%***			

^a^Contribution

For antenatal care utilization; skilled birth attendance (Contr: 43.87%, E_c_: 0.02), television access (Contri: 35.93%, E_c_: 0.47), place of delivery (Contri: 12.37%, E_c_: 0.07) and household wealth (Contri: 106.53%, E_c_: 0.27) were significant positive contributors, while residence status (Contri: − 1.1e+ 02%, E_c_: − 0.32) was a negative significant contributor of maternal health inequalities (Table 4). Women’s education (Contri: 32.53%, E_c_: 0.09), household wealth (Contri: 32.49%, E_c_: 0.27), place of delivery (Contri: 12.05%, E_c_: 0.07), and skilled birth attendance (Contri: 17.98%, E_c_: 0.02) were positive significant drivers of maternal health inequalities (Table 4). The Erreygers decomposing models could not explain 6.94, 0.07% and − 5.90% of variations in maternal health inequalities for SBA, ANC and PNC, respectively (Table 4).

The study extended the decomposition analysis by assessing skilled birth attendance by health personnel. Residence status (Contri: 28.91%, E_c_: − 0.32), women’s education (Contri: 11.54%, E_c_: 0.09) and household wealth (Contri: 35.38%, E_c_: 0.27) were significant contributors of skilled birth attendance inequalities among women assisted by a doctor during delivery (Table 5). While, for women assisted by nurse-midwives at birth; residence status (Contri: 54.72%, E_c_: − 0.32), partner’s education (Contri: 12.30%, E_c_: 0.09), household wealth (Contri: 16.24%, E_c_: 0.27), distance to health facility (Contri: 13.00%, E_c_: − 0.32) and television access (Contri: 51.07%, E_c_: 0.47) were positive drivers of SBA inequalities, and women’s education (Contri: − 23.69, E_c_: 0.09), antenatal care (Contri: − 13.12%, E_c_: 0.03) and postnatal care (Contri: − 13.79%, E_c_: 0.03) were negative drivers of SBA inequalities. Lastly residence status (Contri: 40.34%, E_c_: − 0.32), women’s education (Contri: 14.36%, E_c_: 0.09), partner’s education (Contri: 20.57%, E_c_: 0.09), household wealth (Contri: 37.31%, E_c_: 0.27), distance to health facility (Contri: 70.40%, E_c_: − 0.32) and television access (Contri: − 163.05%, E_c_: 0.47) were significant drivers of skilled birth attendance inequalities.

**Table 5:** Decomposition of skilled birth attendance by health personnel

	***Doctor assisted***				***Nurse-midwife assisted***				***Nurse***			
	Elasticity	Concentration Index	Absolute Contri.	% Contri.	Elasticity	Concentration Index	Absolute Contri.	% Contri.	Elasticity	Concentration Index	Absolute Contri.	% Contri.
Women’s age	0.01	0.01	0.00	0.32	−0.01	0.01	0.00	0.71	0.00	0.01	0.00	0.69
Residence status	−0.05	− 0.32	0.06	28.91	0.05	−0.32	−0.06	54.72	0.01	−0.32	−0.01	40.34
Women’s Education	0.07	0.09	0.03	11.54	−0.07	0.09	−0.02	−23.69	0.01	0.09	0.01	14.36
Partner’ Education	0.06	0.09	0.02	9.65	−0.04	0.09	−0.01	12.30	−0.02	0.09	−0.01	20.57
Household Wealth	0.07	0.27	0.08	35.38	−0.02	0.27	−0.02	16.24	0.01	0.27	0.01	37.31
Household head sex	0.00	−0.02	0.00	0.02	0.00	−0.02	0.00	−0.13	0.01	−0.02	0.00	−1.27
Employment status	0.00	0.12	0.00	0.52	0.00	0.12	0.00	1.87	−0.01	0.12	0.00	12.54
Delivery place	0.03	0.06	0.01	2.92	0.02	0.06	0.00	−3.97	0.02	0.06	0.01	12.63
Antenatal care	0.06	0.03	0.01	3.23	0.11	0.03	0.01	−13.12	0.04	0.03	0.01	13.14
Postnatal care	−0.04	0.03	0.00	−1.82	0.14	0.03	0.01	−13.79	0.04	0.03	0.01	12.48
Distance to facility	0.00	−0.32	−0.01	−2.54	0.01	−0.32	−0.01	13.00	−0.02	− 0.32	0.03	70.40
Radio access	0.00	0.08	0.00	−0.11	−0.01	0.08	0.00	1.57	0.02	0.08	0.01	21.47
Television access	0.00	0.47	0.01	3.21	−0.03	0.47	−0.05	51.07	0.03	0.47	0.06	−163.05
***Residuals***	***8.79%***				***3.21%***				***8.39%***			

## Discussion

This study set out to measure and explain socioeconomic inequalities in maternal healthcare service use in Zimbabwe using the latest available Demographic Health Survey of 2015. Our study findings showed the existence of health-related inequalities in maternal health services uptake (skilled birth attendance, antenatal care, and postnatal care) among women in Zimbabwe across socioeconomic demographic characteristics. The proportion of women who received postnatal care in our study was lower (68%) than what was reported in the 2015 (73%) and there were no significant variances for SBA and ANC, 78 and 76%, respectively [31]. In our study, as expected, maternal services utilization was highest among young women (25-34 years) and significantly low in older women above 44 years. This concurs with what has been observed in other studies across the globe both developed and developing countries [16, 26, 29, 43–59]. Contextually, our study findings on maternal health utilization were consistent with other African studies done in Kenya, Uganda and Ghana [51, 53, 59–68].

This study reported high utilization of skilled birth attendants among rural women, which is contrary to the observed findings from an Ethiopian study that reported more SBA service utilization among urban women [67]. Overall SBA utilization in this study was 93.63%, thus higher than what has been reported in many African developing countries [1, 52, 59, 60]. A Ghanaian study documented that SBA utilization was highest among, women from the poorest households, uneducated, and not attending antenatal care [59]. Conversely, our study revealed SBA utilization to be highest among young employed, educated women from wealthy households who had attended at least four antenatal care visits. The understanding of information is very important in the maternal health [1, 14, 18, 56, 58, 69], thus employed educated women usually have higher socioeconomic status, hence are more likely to utilize maternal health services than uneducated and unemployed women.

Household wealth, place of delivery, and media access have been cited as significant determinants of the antenatal care utilization [51, 60–63]. Our study results showed a negative association between women’s education and utilization of antenatal care services. However, the latter stated findings did not concur with what was observed in Ghana, where women with junior/senior high education were more likely to report antenatal care quality as good [60]. In our study, the distance to health facilities generally influenced women’s perception of antenatal care quality and this increased the relative odds of reporting antenatal quality of care as good to be mainly attenuated by women’s proximity to the health facilities. The aforementioned concurs with findings of a study that focused on antenatal care in sub-Saharan Africa [62].

An Ethiopian study showed that mothers who delivered at a health care facility were more likely to receive PNC than mothers who did not deliver in a health care facility [64]. We observed similar findings as women who delivered at health facilities were more likely to utilize maternal health services (skilled birth attendance, antenatal care, postnatal care). In our study, maternal service (SBA, ANC, PNC) utilization in Zimbabwe was pro-rich, meaning that maternal health utilization favoured women from wealthy households. This was also evident in several other countries [51, 53, 59–68]. This study further computed concentration indices by skilled birth attendant (assisted by; doctor, midwife, nurse). Wealthy women were more likely to be assisted by doctors and nurses, while midwives were more likely to assist poor women. The reason is not clear as to why health inequalities exist between type of health personnel (doctor, nurse, nurse-midwife) that is assisting at birth, and would this imply variations in quality of maternal health care being rendered by doctor or nurse or nurse-midwife? Therefore, more qualitative research to provide more in-depth information should be explored, as decomposing the health inequalities by skilled birth attendants type will only reflect the quantitative aspects of what is driving the health inequalities.

The decomposed results reported household wealth as one of the major drivers of health inequalities in maternal health utilization in Zimbabwe. Our study reported generally high maternal health services usage among rich rural women, which is consistent with other studies in the literature [1, 26, 52, 56, 58, 69]. The better uptake of maternal health services among women with better wealth status can be attributed to their ability to finance the indirect costs (transport costs to health facilities) associated with maternal health services uptake [56]. However, our observed findings underscore the global goals that seek to leave no one behind, as we reported the existence of wealth-related inequalities among women in Zimbabwe. A Ghanaian study also reported socioeconomic status as a significant determinant of skilled birth attendance [59].

The level of education for both the mother and her partner have been cited as important determinants in the uptake of maternal health services in several studies [50, 70, 71]. This was also true in our study, as husband’s education was a significant determinant of skilled birth attendance. Also, woman’s level of education was a significant driver influencing antenatal care attendance. Other studies in the literature have also reported high odds ratios among educated women in relation to antenatal care attendance [57, 63, 72–75]. Our study reported educated women as more likely to use antenatal care services. This might be because educated women are able to exercise autonomy and hold decision-making power compared to uneducated women.

In our study postnatal care service use was relatively high among women who had been assisted by a skilled birth attendant and had at least attended four antenatal care visits. These findings were consistent with a systematic review conducted in Ethiopia [53], however in contrast, our results showed that more rural women attended/utilized postnatal care services compared to urban women. Birth order was an important predictor in explaining the utilization of maternal health services in Zimbabwe, the latter observations maybe due to the uncertainty and the perception of risk associated with first pregnancies. As women were more likely to seek medical attention for first-order births than for subsequent ones [54]. An earlier study conducted in Malawi reported women with a significant high birth order (birth order 2/3) reporting lower likelihood of utilizing PNC compared to women with a first birth order [76].

### Strengths and limitations

Several studies on maternal health in Zimbabwe, have mainly focused on the determinants of maternal health and inequalities in general, however, none to our knowledge have decomposed the socioeconomic inequalities to understand what is driving maternal health inequalities in Zimbabwe. Also, none to our knowledge have used the latest Demographic Health Survey in assessing the current state of maternal health inequalities.

This study had a limitation. The asset index although mostly used in inequality measurement studies, it is sensitive to the assets included in computing the index. Therefore, the main challenge in using asset indicators to measure inequalities is the availability of a sufficiently broad class of asset indicators collected, to allow for differentiation of living standards across all households. Thus, consumption is viewed as “one of the best measures of the economic component of living standards” hence, the preferred unit of analysis for inequality studies in developing countries [77]. However, consumption is not available in the Demographic Health Survey (DHS), the study relied on the best next alternative, which was the asset index. The asset index is generally a good alternative to distinguish socioeconomic layers within the population.

### Policy recommendations

Undoubtedly in many developing countries’ maternal mortality ratios are still very high with huge poor-rich inequalities [43]. Programs targeted to elevate maternal health and reduce maternal mortality often fail to reach women from poor households. In developing countries, maternal socioeconomic inequalities are further exacerbated by the lack of education or low education attainment among women from poor households [1]. Zimbabwe is no exception, hence, the suggestion to improve maternal health inequalities by developing educational policies that target women from poor and socioeconomic deprived households.

Maternal health information should also be provided or disseminated in a form that is easy to understand and accessible, especially poor uneducated women. The explanations of reproductive health issues should be tailored to suite different social contexts, including those with low levels of education and income as education and household wealth were cited as major contributors of health inequalities in Zimbabwe.

As socioeconomic status is one of the major contributors of maternal health inequalities, the scaling up of the maternal voucher program in Zimbabwe, is likely to reduce the inequalities. In 2014, Zimbabwe launched an urban voucher program in Harare and Bulawayo, which aimed at providing pregnant women with access to antenatal care and safe deliveries that they would not otherwise afford [78]. The program was then extended to rural areas under the rural voucher system, to provide access to care for pregnant women and children under-five [78]. The voucher program aimed to increase the demand for maternal health services by increasing their health services quality and giving material subsidies to clinics based on their performance. Therefore, the initial of maternal vouchers are a crucial financial mechanism which can be adopted to improve maternal health access especially among the poor.

## Conclusion

Decomposition analysis showed household wealth, spouse’s education, women’s education and residential status to be important positive contributors of the three (skilled birth attendance, antenatal care and postnatal care) health service utilization outcomes. Therefore, the study suggests that an effective way to reduce the wealth inequality is not only to narrow the gap of income between the rich and poor, but focus on educating women on importance of maternal health services usage.

## Data Availability

All data sets are publicly available on the Demographic Health Survey website at (https://dhsprogram.com/what-we-do/survey/survey-display-406.cfm) and can be accessed upon request from the Demographic Health Survey team.

## Abbreviations

ANC: Antenatal care
CC: Concentration curve
CI: Confidence Interval
E: Erreygers Concentration Index
MDG: Millennium Development Goals
OR: Odds Ratio
SDG: Sustainable Development Goals
SES: Socioeconomic status
SBA: Skilled birth attendance
PNC: Postnatal care
pp: Percentage points
ZDHS: Zimbabwe Demographic Health Survey

## Footnotes

1
*(p < 0.05) means statistically significant at 95% confidence interval*
